# Use of Sensor Imagery Data for Surface Boundary Conditions in Regional Climate Modeling

**DOI:** 10.3390/s110706728

**Published:** 2011-06-28

**Authors:** Hyun Il Choi

**Affiliations:** Department of Civil Engineering, Yeungnam University, 214-1 Dae-dong, Gyeongsan-si, Gyeongbuk-do, 712-749, Korea; E-Mail: hichoi@ynu.ac.kr; Tel.: +82-53-810-2413; Fax: +82-53-810-4622

**Keywords:** sensor imagery, RCM, SBC, GIS

## Abstract

Mesoscale climate and hydrology modeling studies have increased in sophistication and are being run at increasingly higher resolutions. Data resolution sufficiently finer than that of the computational model is required not only to support sophisticated linkages and process interactions at small scales but to assess their cumulative impact at larger scales. The global distributions at fine spatial and temporal scales can be described by means of various senor imagery data collected through remote sensing techniques, sensor image and photo programs, scanning and digitizing skills for existing maps, *etc.* The availability of global sensor imagery maps facilitates assimilation in land surface models to account for terrestrial dynamics. This study focuses on the use of global imagery data for development and construction of surface boundary conditions (SBCs) specifically designed for mesoscale regional climate model (RCM) applications. The several SBCs are currently presented in a RCM domain for the continent of Asia at 30-km spacing by using sensor imagery data. Geographic Information System (GIS) software application tools are mainly used to convert data information from various raw data onto RCM-specific grids. The raw data sources and processing procedures are elaborated in detail, by which the SBCs can be readily constructed for any specific RCM domain anywhere in the world.

## 1. Introduction

The Fourth Assessment Report of the United Nations Intergovernmental Panel on Climate Change (IPCC AR4) [1] has brought to the fore the severity of impacts of global climate changes. In Asia, at its current rate climate change is expected to put close to 50 million extra people at risk of hunger by 2020, rising to an additional 130 million in 2050. Glaciers in the Himalayas could, at a similar rate, disappear altogether by 2035. Further, the IPCC AR4 [1] notes that Europe’s vast reaches of low lying coast are vulnerable to rising sea levels likely to threaten up to 2.5 million people. As global warming and extreme weather pose a severe threat to the safety of life and property over the whole world, climate information from regional climate models (RCMs) has become regarded as a primary tool to address climate and weather variability, changes, and impacts at local and regional scales. Since numerous RCMs have been developed and applied, the next-generation Weather Research and Forecasting (WRF) model (http://www.wrf-model.org/) was developed by a broad community of government and university researchers [2–4]. The WRF was based upon the most advanced supercomputing technologies and promises greater efficiency in computation and flexibility in new module incorporation. The climate extension of the WRF (CWRF) has been developed by the Illinois State Water Survey in collaboration with the WRF Working Groups for incorporating inclusively all WRF functionalities for numerical weather prediction (NWP) while enhancing the capability for climate applications [5].

For all RCMs, one essential component is the representation of surface-atmosphere interactions, which generally requires specification of surface boundary conditions (SBCs). A comprehensive set of SBCs based on best observational data is desired for RCM general applications to all effective, dynamically coupled or uncoupled, combinations of the surface modules as well as portable to any specific region of the world. A critical requirement in constructing the SBCs for the RCM use is that each field must be globally defined with no missing value and physical consistency must be maintained across all relevant parameters. Missing data, if any, must be appropriately filled. The mesoscale weather and climate models, both global and regional, have increased in sophistication and are being run at increasingly higher resolutions. Hence, the raw data should be available at the finest possible resolution and global observation for assimilation in land surface models to improve predictability. The global distributions at fine spatial and temporal scales can be available by various sensor imagery data such as remote sensing observations, sensor photography images, scanned and digitized maps, and so on. This will facilitate a more realistic representation of surface heterogeneity effects. When the data resolution is sufficiently finer than the RCM grid, the subgrid effects can be further incorporated using composite, mosaic or statistical-dynamical approaches [6–10]. With a large volume of global imagery data of the Earth’s terrestrial surface becoming available, precisely monitoring the dynamics of the land surface state variables becomes possible [11].

There is no universal, complete set of SBCs because different modules may require more or less surface parameters to be specified. The input parameter requirement generally depends on the formulation complexity of the surface modules. The CWRF have incorporated into the Common Land Model (CLM) [12], a state-of-the-art model for Soil-Vegetation-Atmosphere Transfer (SVAT). For construction of the primary SBCs in CLM that play an important role in surface-atmosphere interaction, this study has collected many raw datasets at high resolution with global sensor imagery observations freely available. Among the static SBCs, fundamental input fields, independent or defining other derivatives, are orography and vegetation parameters. The surface orography parameters that examine subgrid topography effects on momentum and radiation dynamics include the mean and standard deviation of the terrain elevation at both subgrid-scale and resolved-scale (SOAVE, SOSTD, ROAVE, and ROSTD), and the anisotropy parameter and the angle of the maximum gradient of resolved-scale orography (ROANI and ROANG). The vegetation fields consist of the land cover category (LCC), the fractional vegetation cover (FVC), and the leaf area index (LAI) to determine contribution partitioning between bare soil and vegetation for fluxes crucial to land-atmosphere interactions.

The existing sensor observational databases have various resolutions, a wide range of map projections, and different data formats, and often contain missing values or inconsistencies between variables. This presents significant challenges and requires labor-intensive efforts to process the data onto the RCM-specific grid mesh and input data format. This study employs the Geographic Information System (GIS) software application tools, ArcInfo and ArcMap, from Environmental Systems Research Institute, Inc., particularly to determine the geographic conversion information from a specific map of each raw data to the identical RCM grid system. The information includes location indices, geometric distances, and fractional areas of all input cells contributing to each RCM grid. The grid representative mean values are usually determined by a bilinear interpolation method in terms of the geometric distances and a mass conservative approach as weighted by the fractional areas. For LCC, a categorical field, this study first calculates the total fractional area of each distinct surface category contributing to a given RCM grid and then chooses the dominant one that occupies the largest fraction of the grid.

## 2. General Information

**Figure 1.**
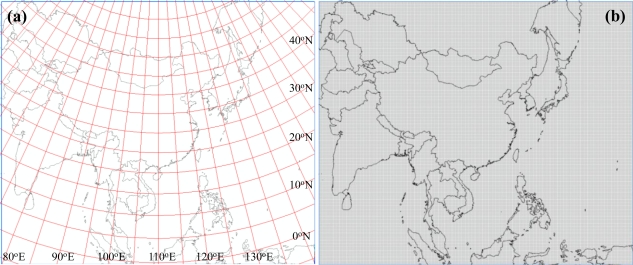
Asia RCM domain overlaid with **(a)** latitude and longitude and **(b)** the 301 × 251 dimensional 30-km spacing grids.

The SBCs data quality, value representation, and visual display largely depend on the RCM computational domain and grid resolution. For climate applications of the Asia region, the domain is centered at 30.0°N and 110.0°W using the Lambert Conformal Conic map projection and 30-km horizontal grid spacing, with total grid numbers of 301 (west-east) × 251 (south–north). Figure 1(a) shows the country map overlaid with latitude and longitude lines projected on the Lambert Conformal Conic map, and Figure 1(b) denotes the 301 × 251 dimensional 30-km spacing grids over the Asia RCM domain. The domain covers the most parts of Asia and represents the regional climate that results from interactions between the planetary circulation and surface processes, including orography and vegetation fields. In this study, the fundamental SBCs using sensor imagery observations are constructed and displayed on this RCM domain, suitable for Asia applications.

For convenience, the geographic location of a point is hereafter referred as a “pixel” for raw data and a “grid” for the RCM result. A given pixel or grid value represents the area surrounding the point as defined by its respective horizontal spacing. The following section elaborates in detail on raw data sources and processing procedures used to construct any specific RCM domain over the globe. Most procedures use ArcInfo and ArcMap commands. In particular, IMAGEGRID and GRIDPOLY convert input data from the sensor image to the ArcGIS raster grid and to the polygon coverage formats, respectively; PROJECT remaps the raw input data onto the RCM grid projection; UNION and CLIP geometrically intersect polygon features of input data with the RCM grid mesh and extract the fractional area of each pixel contributing to the grid; GRID DOCELL and IF statements conditionally merge, replace, or adjust different input datasets for an improved product. Even relatively finer resolution (8-km) input data, LAI, has missing value pixels due to cloud contamination and atmospheric effects, which are filled by the spatial average over the nearby data pixels having the same land cover category within a certain radius around a missing point. The number of pixels and the range of radius used for filling depend on the resolution of the raw input data. Since the 30-km LAI for the RCM are generated from the two different resolution data of the 1-km FVC and the 8-km LAI, the 1-km data are integrated onto the 8-km map and then a smoothing filter is introduced to remove abnormal values due to inconsistency of the two data at individual pixels (see Section 3.4 for details).

## 3. Surface Boundary Conditions

The various surface orography parameters (SOAVE, SOSTD, ROAVE, ROSTD, ROANI, and ROANG) have been introduced for use of analysis on orographic turbulence effects under stable atmospheric conditions by using the terrain elevation model through the multi sensor imagery dataset. The vegetation characteristic fields (LCC, FVC, and LAI) for use of prediction on fluxes crucial to land-atmosphere interactions have been constructed by using satellite remote sensing data. The details about the raw data sources and processing procedures for each SBCs for RCM uses in the Asia domain are discussed below.

### 3.1. Surface Orography Parameters

These fields are constructed from the U.S. Geological Survey (USGS) HYDRO1k Digital Elevation Model (DEM) with a 1-km nominal cell size (http://eros.usgs.gov/#/Find_Data/Products_and_Data_Available/HYDRO1K), which is developed at the U.S. Geological Survey’s Earth Resources Observation and Science (EROS) Data Center to provide to users, on a continent by continent basis, hydrologically correct DEMs for use in continental and regional scale modeling and analyses. It is based on the Global 30-arc-second elevation dataset (GTOPO30) derived from several different raster and vector sources of topographic information. The GTOPO30 data were produced jointly by several national and international organizations. National Imagery and Mapping Agency (NIMA) produced Digital Terrain Elevation Data (DTED) which was used for the source of most parts of Eurasia in GTOPO30. DTED is a raster topographic data collected through remote sensing techniques, aerial image sensor photography, digitization of interpolated contour lines on existing maps, and actual on-site surveying measurements. The DEM data is available in the band interleaved by line (BIL) image format on the Lambert Azimuthal equal area projection. For construction of surface orography parameters, the raw imagery data are converted into ArcGIS raster grid format and then remapped onto the RCM projection. Subsequently, the ArcInfo/GRID commands, ZONEALMEAN and ZONALSTD, are used to calculate the mean and standard deviation of the subgrid-scale elevations, SOAVE and SOSTD, respectively, within each RCM grid. The mean and deviation from the centroid of each grid are picked up by ArcInfo’s Arc Macro Language (AML) program Gridspot70 for SOAVE and SOSTD.

**Figure 2.**
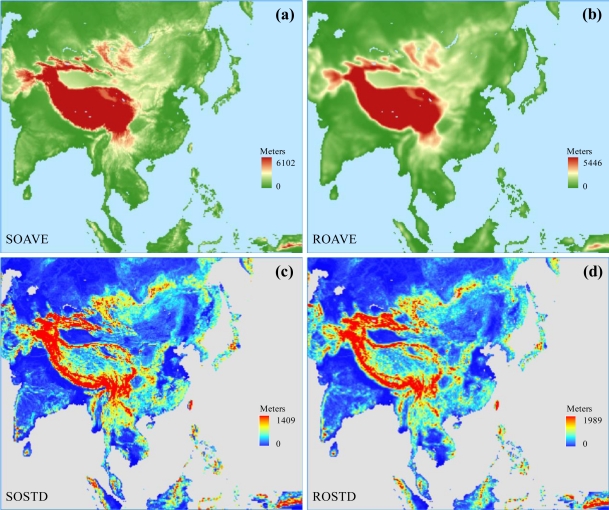
The comparison of geographic distributions of surface orography parameters over the Asia RCM domain. **(a)** and **(b)** denote mean terrain elevations at subgrid-scale and resolved-scale, respectively; **(c)** and **(d)** denote standard deviations of terrain elevations at subgrid-scale and resolved-scale, respectively.

Given a grid spacing *Δx*, the subgrid variability effects of topography are currently not accounted in most RCMs. Subgrid orography effects need to be incorporated in RCMs through certain parameterizations especially in mesoscale global models. The values of the surface orography parameters depend crucially on the resolution of the raw topography data derived for a target model grid that has a lower resolution than the source data pixel. It is necessary to filter the subgrid-scale orography variables in a proper scale to avoid numerical noise for the model integrations. For the numerical stability the terrain height is filtered to remove 4-grid waves, and the subgrid effects are denoted based on the mean terrain averaged over an area of (*4Δx*)^2^ surrounding each grid center. Thus the resolved mesoscale orographic parameterizations are here calculated from the terrain elevation (*h_i_* in meters) by the HYDRO1k DEM with a 1-km nominal cell size. Following Rontu [13], the resolved resolved-scale mean elevation, ROAVE (*H_4Δx_* in meters), is calculated for 4 grid-lengths (*4Δx*) using double smoothing method:

(1) $$H_{4\text{Δ}x}\;=\;\frac{1}{N_{4\text{Δ}x}}\;\sum_{i=1}^{N_{4\text{Δ}x}}h_{9i}$$

where $h_{9i}=\frac{1}{9}\sum_{i=1}^{9}h_{i}$ (in meters) is 9-point averaged elevation including the point and its 8 neighbor points based on 1-km DEM (*h_i_*), *Δx* is the RCM grid-length which is 30 km for the current model, and *N_4Δx_* is the number of 1-km DEM pixels within a *4Δx*-resolution grid. As defined in Rontu [13], the standard deviation of resolved-scale elevations, ROSTD (*σ_m_* in meters), for the difference between *h_9i_* and *H_4Δx_* is calculated as:

(2) $$\sigma_{m}=\sqrt{\frac{1}{N_{\Delta x}-1}\sum_{i=1}^{N_{\Delta x}}\zeta^{2}}$$

where *ζ* = *h_9i_* – *H*_*4*Δ*x*_ is *h_9i_* departure from *H*_*4Δx*_, and *N_Δx_* is the number of 1-km data points for each *Δx*-resolution grid. Figure 2 compares geographic distributions of mean terrain elevations, and standard deviations of terrain elevations at subgrid-scale and resolved-scale.

Other parameters for resolved-scale orography are anisotropy, ROANI (*α*), characterizing the anisotropy of orography, and direction angle, ROANG (*θ*), representing the mean slope within each grid. The parameters ROANI (*α*) and ROANG (*θ*) are based on the orographic gradient correlation tensor by Lott and Miller [14]:

(3) $$H_{ij}=\bar{\frac{\partial \zeta }{\partial x_{i}}\frac{\partial \zeta }{\partial x_{j}}}$$

where *x_i_* and *x_j_* are the two principle coordinates of the model grids. Note that the parameters with overbar (hereafter) are model grid average values. Following Rontu *et al*. [15], the two intermediate parameters are defined respectively as:

(4) $$T=\bar{\frac{\partial \zeta }{\partial x_{i}}\frac{\partial \zeta }{\partial x_{i}}}+\bar{\frac{\partial \zeta }{\partial x_{j}}\frac{\partial \zeta }{\partial x_{j}}}$$

(5) $$D=\sqrt{{\left(\bar{\frac{\partial \zeta }{\partial x_{i}}\frac{\partial \zeta }{\partial x_{i}}}-\bar{\frac{\partial \zeta }{\partial x_{j}}\frac{\partial \zeta }{\partial x_{j}}}\right)}^{2}+4{\left(\bar{\frac{\partial \zeta }{\partial x_{i}}\frac{\partial \zeta }{\partial x_{j}}}\right)}^{2}}$$

Thus, the anisotropy ROANI (*α*) is finally calculated as:

(6) $$\alpha =\sqrt{\frac{\left|T-D\right|}{\left|T+D\right|}}$$

The anisotropy is zero for a two-dimensional ridge and increases for circular-shaped mountains. The direction angle ROANG (*θ* in radian) between the maximum gradient of resolved-scale orography and the *x_i_* -axis of the model grid is defined as:

(7) $$\theta =\text{arctan}\left(\frac{\bar{\frac{\partial \zeta }{\partial x_{j}}\frac{\partial \zeta }{\partial x_{j}}}-\bar{\frac{\partial \zeta }{\partial x_{i}}\frac{\partial \zeta }{\partial x_{i}}}+D}{2\bar{\frac{\partial \zeta }{\partial x_{i}}\frac{\partial \zeta }{\partial x_{j}}}}\right)$$

**Figure 3.**
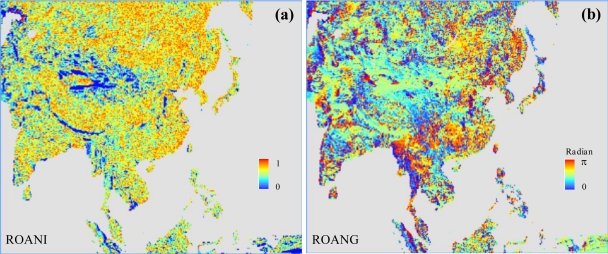
The geographic distributions of **(a)** anisotropy and **(b)** direction angle at resolved-scale over the Asia RCM domain.

Figure 3 illustrates the geographic distributions of anisotropy and direction angle over the Asia RCM domain.

### 3.2. Land Cover Category

The RCM uses the 24-category USGS land cover classification (see Table 1) developed from the April 1992–March 1993 Advanced Very High Resolution Radiometer (AVHRR) satellite-derived Normalized Difference Vegetation Index (NDVI) composites. This data is based on a flexible data base structure and seasonal land cover regions concepts. The regions are composed of relatively homogeneous land cover associations which exhibit distinctive phenology, and have common levels of primary production. The raw data are available at 1-km spacing on the geographic coordinate system in BIL image format (http://edc2.usgs.gov/glcc/glcc.php), converted into the ArcGIS raster grid and polygon coverage, and remapped onto the RCM projection. The fractional area of each pixel contributing to the grid is extracted after the result is intersected with the RCM grid mesh. The contributing area for each of the 24 LCCs is summed over all pixels of the same category within each RCM grid. The category contributing the largest area is chosen as the LCC for the grid. When the fractional area of water bodies is less than 0.5 but dominates the grid, the category chosen is the one contributing the second largest area.

**Figure 4.**
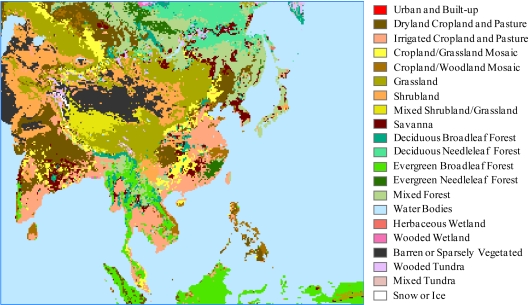
The geographic distribution of 21 LCC categories over the Asia RCM domain.

Figure 4 illustrates the LCC geographic distribution over the RCM domain. Note that the USGS raw data do not contain LCC types 4 (mixed dryland/irrigated cropland & pasture) and 20 (herbaceous tundra) over the globe, and additionally LCC type 23 (bare ground tundra) is not chosen for LCC majority category in the Asia RCM domain. Therefore, the final LCC includes only 21 categories of LCCs over the present RCM domain.

### 3.3. Fractional Vegetation Cover

The FVC is one ecological parameter that determines contribution partitioning between bare soil and vegetation for surface evapotranspiration, photosynthesis, albedo, and other fluxes crucial to land-atmosphere interactions. Following Zeng *et al*. [16,17], the time-invariant FVC is derived from the 10-day April 1992–March 1993 composites of the global 1-km AVHRR NDVI product. The annual maximum NDVI (*N_p,max_*) for each LCC are chosen to minimize the effect of cloud contamination on data quality. For each pixel, the vegetation cover is computed by:

(8) $$C_{v}=\frac{N_{p,\text{max}}-N_{s}}{N_{c,v}-N_{s}}$$

where *N_c,v_* is the NDVI value for a complete coverage of a specific USGS LCC over the pixel and *N_s_* for bare soil.

Using a commercial imagery database, Zeng *et al*. [16] determined *N_c,v_* by examining percentiles of the *N_p,max_* histogram for each LCC of the International Geosphere Biosphere Programme (IGBP) classification [18,19]. Liang *et al*. [20] calculated the *N_c,v_* values for the 24 USGS LCCs from those of the 17 IGBP categories by intersecting the USGS and IGBP land cover maps and computing the fractional areas of individual IGBP categories contributing to each USGS category. The final *N_c,v_* is the average of all contributing IGBP values weighted by their corresponding fractional areas. Corresponding *N_c,v_* values and occupation rates for the USGS and IGBP categories are listed in Table 1.

**Figure 5.**
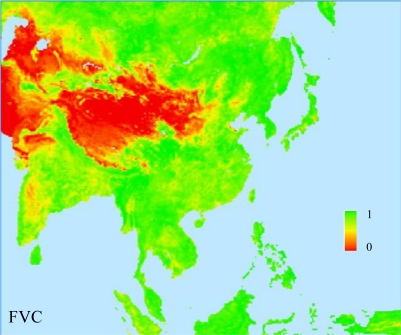
The geographic distributions of FVC derived from the April 1992–March 1993 AVHRR over the Asia RCM domain.

The resultant *C_v_* point data at 1-km spacing are converted to polygon coverage data, remapped onto the RCM projection, and intersected with the RCM grid mesh. The fractional area of each pixel contributing to the grid is extracted. The final FVC is obtained by the area-weighted averaging of *C_v_* values for all pixels within each RCM grid. Figure 5 illustrates the FVC geographic distributions derived from the AVHRR NDVI over the Asia RCM domain.

### 3.4. Leaf Area Index

The LAI is defined as the total one-sided area of all green canopy elements over vegetated ground area, which are constructed from the global monthly mean distributions of green vegetation leaf area index, based on the July 1981–December 1999 AVHRR NDVI data at 8-km spacing on the Interrupted Goode Homolosine projection provided by Boston University [21,22]. LAI has missing value pixels due to cloud contamination and atmospheric effects, which are filled by the spatial average over nearby data pixels having the same LCC within a certain radius starting from 16 km (24 pixels) around a missing point and increasing until a 3-pixel minimum is obtained. Filled data are converted into the raster grid, then the polygon coverage, and remapped onto the RCM projection. After this result is further adjusted to be confined by the USGS LCC (see Section 3.2) for a consistent representation of water bodies, the adjusted raw data (*L_raw_*) with respect to unit ground area is divided by local vegetation cover *C_v_* to define the green leaf area index (*L_gv_*) with respect to vegetated area only following Zeng *et al*. [17]. There is inconsistency between *C_v_* and *L_raw_* data at individual pixels mainly because *C_v_* was derived based on the 24 USGS LCCs at 1-km spacing, but *L_raw_* in terms of six alternative biomes with distinct vegetation structures at an 8-km interval. For the RCM, the 1-km *C_v_* data are integrated onto the 8-km *L_raw_* map to compute *L_gv_* guess values and then a smoothing filter is introduced to remove abnormal values due to inconsistency between *C_v_* and *L_raw_* data at individual pixels. Liang *et al*. [20] designed the filter through trial and error by examining the frequency distribution of abnormal *L_gv_* values and considering the canopy displacement height in the CLM for each USGS LCC. The point value that exceeds the filter threshold listed in Table 2 is filled by the average over nearby data pixels having the same LCC within a certain radius starting from 16 km (24 pixels) around the point and increasing until a 3-pixel minimum is obtained.

In addition, *L_gv_* data contain large uncertainties in winter due to cloud contamination, especially for the USGS LCC types 13 and 14 (evergreen broadleaf and needleleaf forests). Following Zeng *et al*. [17], *L_gv_* values in winter months for these two categories are adjusted by:

(9) $$L_{gv}=\text{max}(L_{gv},cL_{gv,\text{max}})$$

where correction coefficient *c* is 0.8 and 0.7 for LCC types 13 and 14 respectively, and *L_gv,max_* is the maximum *L_gv_*. For the climatology, the maximum can be determined from all monthly values during the entire period, while for interannual variations it is taken in three consecutive years.

**Figure 6.**
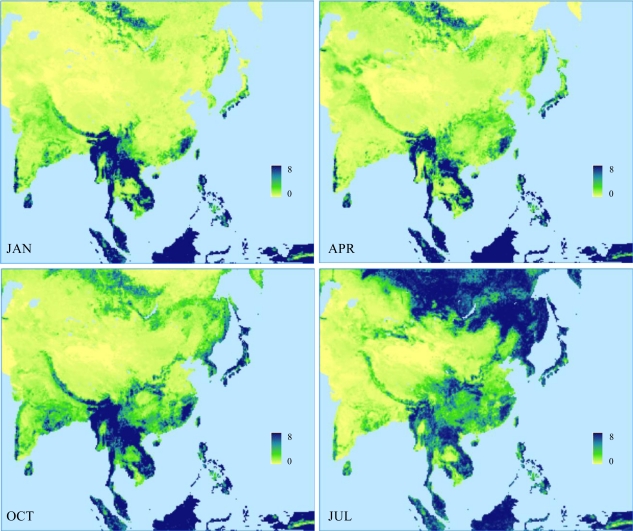
The geographic distributions of mean LAI based on the original 1981–1999 AVHRR climatology data in January, April, July, and October over the Asia RCM domain.

After extreme value removal and winter adjustment at each 8-km pixel, the new *L_gv_* is multiplied by its respective *C_v_* and then intersected with the RCM grid mesh. The fractional area of each pixel contributing to the grid is extracted. The area-weighted averaging of all pixels within each RCM grid results in the new LAI per unit ground, which will be divided by local FVC (see Section 3.3) to produce the final LAI. Figure 6 depicts January, April, July, and October mean LAI distributions of the AVHRR climatologies over the RCM domain.

## 4. Conclusions

The increase in resolution of regional climate and mesoscale atmospheric models can be facilitated by availability of sensor imagery observations in global distributions at the finest spatial and temporal scales. A large volume of global sensor imagery data of the Earth’s terrestrial surface can improve model predictability by supporting better model parameterizations for dynamics of the land surface-atmosphere interactions and increasingly sophisticated assimilation schemes in land surface models. This study focuses on the construction of fundamental SBCs desired for general RCM applications to all effective, dynamically coupled or uncoupled, combinations of the surface modules, as well as portability to any specific region of the world. The new SBCs development motivated by the limitations and inconsistencies of existing SBCs can be readily incorporated into any RCM suitable for climate and hydrology modeling studies. The primary SBCs constructed by using sensor imagery data include surface orography parameters such as means and deviations of terrain elevation at both subgrid-scale and resolved-scale, along with anisotropy and direction angle, and vegetation parameters such as land cover category, fractional vegetation cover, and leaf area index. A critical requirement in constructing the SBCs for RCM use is that each variable must be defined globally with physical consistency across all relevant parameters. This study tried to appropriately manage and rectify existing databases that have various resolutions, a wide range of map projections, different data formats, and often contain missing values or inconsistencies between variables. The GIS application tools such as ArcInfo and ArcMap are mainly used to process vast amount of raw data and to determine the geographic conversion information from a specific map projection of raw data to the identical RCM grid system. Given that data quality and value representation depend on the RCM computational domain and grid resolution, all the SBCs are constructed onto the 30-km RCM domain suitable for the continent of Asia. The raw data sources and processing procedures for the SBCs can be readily used for any specific RCM domain in the world. Even for choosing the best-available data quality, comprehensive processing procedures, and consistency between alternatives, the SBCs so constructed carry over uncertainties inherent in the raw data. Future studies will be required to assess impacts of the SBCs treatments, and an upcoming paper will address the RCM climate sensitivity to these SBCs.

## Figures and Tables

**Table 1.** Comparison of *N_c,v_* between USGS and IGBP Land Cover Legends.

**USGS land use/land cover legend**			**IGBP land cover legend**			
Type	Description	*N*_*c*,*v*_	Type	Description	*N_c,v_*	Occupation ratio (%)
1	Urban and Built-Up Land	0.62	13	Urban and Built-Up	0.62	100
2	Dryland Cropland and Pasture	0.61	12	Croplands	0.61	100
3	Irrigated Cropland and Pasture	0.61	12	Croplands	0.61	94.41
			14	Cropland/Natural Vegetation Mosaic	0.65	5.59
5	Cropland/Grassland Mosaic	0.65	12	Croplands	0.61	1.33
			14	Cropland/Natural Vegetation Mosaic	0.65	98.67
6	Cropland/Woodland Mosaic	0.65	14	Cropland/Natural Vegetation Mosaic	0.65	100
7	Grassland	0.49	10	Grasslands	0.49	100
8	Shrubland	0.60	6	Closed Shrublands	0.60	14.81
			7	Open Shrublands	0.60	81.00
			8	Woody Savannas	0.62	4.19
9	Mixed Shrubland/Grassland	0.59	6	Closed Shrublands	0.60	15.28
			7	Open Shrublands	0.60	76.53
			10	Grasslands	0.49	8.19
10	Savanna	0.60	8	Woody Savannas	0.62	50.48
			9	Savanna	0.58	49.52
11	Deciduous Broadleaf Forest	0.70	2	Evergreen Broadleaf Forest	0.69	20.41
			4	Deciduous Broadleaf Forest	0.70	68.26
			5	Mixed Forest	0.68	11.33
12	Deciduous Needleleaf Forest	0.63	4	Deciduous Needleleaf Forest	0.63	100
13	Evergreen Broadleaf Forest	0.69	2	Evergreen Broadleaf Forest	0.69	100
14	Evergreen Needleleaf Forest	0.63	1	Evergreen Needleleaf Forest	0.63	100
15	Mixed Forest	0.68	5	Mixed Forest	0.68	100
16	Water Bodies		17	Water Bodies		100
17	Herbaceous Wetland	0.56	11	Permanent Wetlands	0.56	100
18	Wooded Wetland	0.56	11	Permanent Wetlands	0.56	100
19	Barren or Sparsely Vegetated	0.60	16	Barren or Sparsely Vegetated	0.60	100
21	Wooded Tundra	0.61	7	Open Shrublands	0.60	68.11
			8	Woody Savannas	0.62	31.89
22	Mixed Tundra	0.60	16	Barren or Sparsely Vegetated	0.60	100
23	Bare Ground Tundra	0.60	16	Barren or Sparsely Vegetated	0.60	100
24	Snow or Ice		15	Snow and Ice		100

Note: Land cover types 4 (Mixed Dryland/Irrigated Cropland & Pasture) and 23 (Herbaceous Tundra) do not exist in the global dataset.

**Table 2.** Parameters in deriving LAI for each USGS Land Cover.

**Type**	**USGS land cover legend**	**FVC (1 km)**	**Displacement height (m)**	***Lgv* filter threshold**
1	Urban and Built-Up Land	0.735	0.667	7
2	Dryland Cropland and Pasture	0.875	0.667	7
3	Irrigated Cropland and Pasture	0.804	0.667	7
5	Cropland/Grassland Mosaic	0.729	0.667	7
6	Cropland/Woodland Mosaic	0.869	0.667	7
7	Grassland	0.711	0.667	6
8	Shrubland	0.381	0.333	5
9	Mixed Shrubland/Grassland	0.391	0.333	5
10	Savanna	0.848	0.667	7
11	Deciduous Broadleaf Forest	0.871	13.333	8
12	Deciduous Needleleaf Forest	0.920	13.333	8
13	Evergreen Broadleaf Forest	0.953	23.333	8
14	Evergreen Needleleaf Forest	0.895	13.333	8
15	Mixed Forest	0.875	13.333	8
16	Water Bodies	–	0.667	–
17	Herbaceous Wetland	0.947	13.333	6
18	Wooded Wetland	0.835	0.667	8
19	Barren or Sparsely Vegetated	0.073	0.333	4
21	Wooded Tundra	0.714	0.667	6
22	Mixed Tundra	0.323	0.333	6
23	Bare Ground Tundra	0.018	0.333	6
24	Snow or Ice	–	0.667	–

Note: Land cover types 4 (Mixed Dryland/Irrigated Cropland & Pasture) and 23 (Herbaceous Tundra) do not exist in the global dataset.
